# Do we really ponder about necessity of intravenous hydration in acute bronchiolitis? 

**Published:** 2016-03-30

**Authors:** Şule Yıldırım, Nazan Kaymaz, Naci Topaloğlu, Fatih Köksal Binnetoğlu, Mustafa Tekin, Hakan Aylanç, Fatih Battal, Burçin Gön&uuml;ll&uuml;

**Affiliations:** Çanakkale Onsekiz Mart University, Faculty of Medicine, Department of Pediatrics, Çanakkale, Türkiye

**Keywords:** Bronchiolitis, hospitalization, fluid therapy, patient care management

## Abstract

**Objective:**

The goal was to establish the role of intravenous hydration therapy on mild bronchiolitis.

**Methods:**

This was a retrospective case control study. Infants between 1 month and 2 years of age admitted to our general pediatrics ward between June 2012 and June 2013 with a diagnosis of uncomplicated acute bronchiolitis were enrolled to the study. Hospital medical files were reviewed to get information about children personal history, symptoms of the disease, disease severity scores and their management. Patients were classified into 4 groups according to the management; nebulized short-acting β_2_-agonist (salbutamol) +hydration; nebulized short-acting β_2_-agonist (salbutamol); hydration and neither bronchodilator nor hydration. We examined length of stay in the hospital as an outcome measure.

**Results:**

A total of 94 infants were studied. There was no significant difference between groups in terms of length of stay in hospital.

**Conclusions:**

IV hydration is not effective on length of stay in hospital in mild acute bronchiolitis patients.

## Abstract (es)

**Objetivo:**

Establecer la función de la terapia de hidratación intravenosa leve.

**Métodos:**

Estudio retrospectivo de casos y controles. En el estudio fueron reclutados niños entre 1 mes y 2 años de edad atendidos en la sala de pediatría general entre junio 2012 y junio 2013, con diagnóstico de bronquiolitis aguda no complicada. Se revisaron historias médicas de los niños para obtener datos personales, síntomas de la enfermedad, grado de severidad y el manejo instaurado. Los pacientes fueron clasificados en cuatro grupos de cuerdo al manejo: hidratación + nebulización de corta acción con β_2_-agonista (salbutamol); nebulización de corta acción con β_2_-agonista (salbutamol); hidratación; o sin hidratación y broncodilatador. Se determinó la duración de la estancia hospitalaria como medida resultado.

**Resultados:**

Un total de 94 niños fueron estudiados. No hubo diferencia significativa entre los grupos en términos de duración de la estancia en el hospital.

**Conclusiones:**

La hidratación IV no es efectiva en la duración de la estancia hospitalaria en pacientes con bronquiolitis aguda leve.

## Introduction

Acute bronchiolitis is one of the most common diseases of childhood, characterized by inflammation, edema, necrosis of small airways and bronchospasm. The typical symptoms are cough, rhinitis, tachypnea, wheezing and respiratory distress 1,2. The main principles of treatment are supportive including follow up of oxygen saturation, fluid balance and nutrition status 3. There are diverse variations in diagnosis and treatment of acute bronchiolitis in children. Also there is not a consensus about the fluid therapy in acute bronchiolitis. It is shown that personal preferences of physicians and hospitals are more important than the severity of the disease in management 4-6. Therefore, guidelines for management of acute bronchiolitis have been established around the world 7,8. One the most important guideline was reported by American Academy of Pediatrics (AAP) 7. It was emphasized that clinicians should evaluate the hydration status and the oral intake of the patient. According to the AAP guideline if the oral intake of the infant is not affected, only close observation is adequate. Intravenous hydration is required only if oral intake is not safe. Moreover, over hydration especially in sick infant can cause syndrome of inappropriate antidiuretic hormone secretion. Podder *et al*. 9, showed that water retention commonly accompanies acute bronchiolitis and they claimed that fluid therapy may cause water intoxication. However, applying IV route and IV hydration is readily and routinely done in most clinics without evaluating the hydration status of the infant. 

We hypothesized that unnecessary IV hydration is not effective on treatment of acute bronchiolitis. In our center, six different general pediatricians follow the patients which may lead to different approaches about IV hydration in children with bronchiolitis. Infants hospitalized with acute bronchiolitis were reviewed retrospectively to observe the effect of hydration on the length of hospital stay.

## Materials and Methods

### Study design

This was a retrospective case control study. The Research Ethics Committee of the Çanakkale Onsekiz Mart University approved the study. 

### Study population

Infants between 1 month and 2 years of age admitted to our hospital general pediatrics ward with a diagnosis of uncomplicated acute bronchiolitis were enrolled. Inclusion criteria were; 1- the primary diagnosis of acute bronchiolitis; 2- mild and moderate disease according to the Wang-clinical score 5. Exclusion criteria were; having a chronic disease, vomiting preventing oral feeding, severe and complicated disease, and recurrent disease. 

### Data collection

Personal history, symptoms of the disease, disease severity scores and their management were recorded from the hospital database and chart system retrospectively. We examined length of stay (LOS) in the hospital as an outcome measure. Discharge from hospital was once infants had attained stable SpO_2_ of 94% or higher continuously for 4 h (including a period of sleep) and were feeding orally at 75% or more of their expected intake of milk daily.

According to the treatment regimens patients were classified into 4 groups, all patients had supportive management (nutrition, oxygen, monitorization);

Group 1: nebulized short-acting β_2_-agonist (salbutamol) +hydration

Group 2: nebulized short-acting β_2_-agonist (salbutamol)

Group 3: hydration

Group 4: neither bronchodilator nor hydration

### Statistical analysis

Data were analyzed using the Statistical Package for the Social Sciences (SPSS^®^) Version 16.0 (Chicago, IL). Descriptive data were presented as number and percentage. Normality of distribution was done with analytical tests. The variables were found not to be normally distributed and Kruskal-Wallis test was used for comparisons of groups. Mann Whitney U test was used to compare independent two groups. Comparison between categorical indicators was performed using the χ^2^ test. *p* values less than 0.05 was assumed as significant. 

##  Results

### Sociodemographic characteristics

There were 147 admissions with the diagnosis of acute bronchiolitis. 53 patients were excluded for the following reasons: 7 had chronic diseases, 14 had severe bronchiolitis, 11 had vomiting and 21 were recurrent admission. A total of 94 infants were included. The average age of children was 15.37±6.85 months. Sixty-five of them were boys and 29 were girls. Patients were classified into 4 groups according to the management. Demographic characteristics of the patients in different treatment groups were summarized in Table 1. 

**Table 1.** Demographic characteristics of the patients in different treatment groups.

Variable	Group 1 (n= 25)	Group 2 (n= 20)	Group 3 (n= 20)	Group 4 (n= 29)	p *
Age (month)	13.44±7.44	14.95±7.04	16.90±6.44	15.93±6.41	0.269
Sex n(%)					
Girl	4 (16)	8 (40)	9 (45)	8 (27.6)	0.145
Boy	21 (84)	12 (60)	11 (55)	21 (72.4)	
Gestational age (week)	39.04±1.17	39.05±1.28	38.90±1.29	39.21±1.26	0.851
Delivery type n (%)					
Cesarian section	11 (44)	9 (45)	10 (50)	13 (44.8)	0.979
Normal vaginal	14 (56)	11 (55)	10 (50)	16 (55.2)	
Nutritional status n (%)					
Only breastfeeding	3 (12.0)	2 (10.0)	1 (5.0)	-	0.675
Breastfeeding + complemetary feeding	9 (36.0)	8 (40.0)	8 (40.0)	11 (37.9)	
Only complementary feeding	13 (52.0)	10 (50.0)	11 (55.0)	18 (67.1)	
LOS (day)	4.32±1.28	4.25±1.33	4.30±1.03	4.10±1.01	0.914

^* ^Kruskal-wallis test

n: number

LOS: length of stay

Comparison of group 1 vs group 2 and group 3 vs group 3 did not reveal a significant difference between gestational age, current age, delivery type and feeding properties (Table 1). 

### Length of hospital stay in the groups

Mean LOS was 4.23±1.15 days for all patients. There was no significant difference between groups in terms of LOS in hospital (Table 1). Similarly, when the four study groups were compared two by two, LOS was not different between groups 1 and 2 (4.32±1.28 vs 4.25±1.33, *p*= 0.794). Similarly, LOS was not different between groups 3 and 4 either (4.30±1.03 vs. 4.10±1.01,* p*= 0.469). 

## Discussion

The results of our study suggest that routine IV hydration is not effective on decreasing LOS in hospital in mild acute bronchiolitis patients. 

The main factor in the treatment of acute bronchiolitis is a good follow-up and supportive care. Respiratory distress, apnea and hypoxia parameters should be monitored closely. Although there is not a standardized treatment, supportive care should aim to treat hypoxia and respiratory failure. Therefore it is important to closely monitor the respiratory distress findings, feeding status and temperature of the infant.

Respiratory rate is one of the most important factors that determine the infant's intravenous access requirement. Other parameters requiring intravenous access are severity of the disease, persistent vomiting and decreasing saturation during feeding despite oxygen treatment. However re-initiation of oral feeding as soon as possible is the recommended management. 

Preservation of hydration status in infants is essential in management of most diseases. Acute and chronic fluid deficits may adversely affect vulnerable populations' especially young children 10. Johnson *et al*. 11, stated that 30% of children with acute bronchiolitis need hydration because acute bronchiolitis cause inadequate feeding, increased respiratory effort and dehydration due to fever. However, there is not a consensus in which statements or ways hydration therapy should be used. Hydration can be assessed by IV route or nasogastric (NG) tube. Babl *et al*. 12, reported that in pediatric emergency units of tertiary centers in Australia and New Zeeland 48% of infants had NG and 52% of infants had IV fluid replacement. It is reported that IV therapy has not more benefit over NG rehydration although NG way is regarded as more unpleasant and invasive by some practitioners 13 14.

IV hydration is used commonly in acute bronchiolitis. However, it can cause fluid overload and electrolyte imbalance 15. It was shown that in 30% of children that had IV hydration hyponatremia had developed 16. Fluid overload may also lead to pulmonary congestion 17. IV catheterization is a common but unpleasant experience in children especially during hospitalization period 18. Moreover children can become anxious with IV route and can refuse oral feeding. The other complication with over hydration especially in sick infant is inappropriate antidiuretic hormone secretion. Furthermore, AAP guidelines do not recommend routine use of bronchodilators 7. Johnson *et al*. 19, showed no reduction in use of bronchodilators after guidelines. In our study we observed that bronchodilator usage rate was 47.8%. However, it was not effective on LOS in mild bronchiolitis in consistence with the literature. 

## Conclusion

In conclusion, we could not show the beneficial effect of IV hydration on LOS in hospitalized infants with the diagnosis of acute mild bronchiolitis. Only supportive care was effective. Therefore, physicians should evaluate the patients' symptoms and signs carefully; the IV hydration should not be routine procedure to perform immediately.
